# A global test for gene‐gene interactions based on random matrix theory

**DOI:** 10.1002/gepi.21990

**Published:** 2016-07-07

**Authors:** H. Robert Frost, Christopher I. Amos, Jason H. Moore

**Affiliations:** ^1^ Department of Biomedical Data ScienceGeisel School of Medicine, Dartmouth CollegeHanoverNew HampshireUnited States of America; ^2^ Division of InformaticsDepartment of Biostatistics and EpidemiologyInstitute for Biomedical InformaticsPerelman School of Medicine, University of PennsylvaniaPhiladelphiaPennsylvaniaUnited States of America

**Keywords:** gene‐gene interaction, random matrix theory, global test

## Abstract

Statistical interactions between markers of genetic variation, or gene‐gene interactions, are believed to play an important role in the etiology of many multifactorial diseases and other complex phenotypes. Unfortunately, detecting gene‐gene interactions is extremely challenging due to the large number of potential interactions and ambiguity regarding marker coding and interaction scale. For many data sets, there is insufficient statistical power to evaluate all candidate gene‐gene interactions. In these cases, a global test for gene‐gene interactions may be the best option. Global tests have much greater power relative to multiple individual interaction tests and can be used on subsets of the markers as an initial filter prior to testing for specific interactions. In this paper, we describe a novel global test for gene‐gene interactions, the global epistasis test (GET), that is based on results from random matrix theory. As we show via simulation studies based on previously proposed models for common diseases including rheumatoid arthritis, type 2 diabetes, and breast cancer, our proposed GET method has superior performance characteristics relative to existing global gene‐gene interaction tests. A glaucoma GWAS data set is used to demonstrate the practical utility of the GET method.

## 1 INTRODUCTION

Interactions between markers of genetic variation, or gene‐gene (G× G) interactions, are thought to have an important biological role in many polygenic diseases and other complex phenotypes (Moore, 2003; Cordell, 2009; Taylor & Ehrenreich, 2015; Mackay, 2014). Although such interactions can be defined as either statistical interactions relative to a specific model or as qualitative, biological associations, we focus solely on the former type of interaction in this paper (see Section 2.1.5 below for the exact statistical model). Knowledge of biologically valid gene‐gene interactions has the potential to improve our understanding of the genetic regulation of cellular processes, explain a greater proportion of the known heritability of human diseases, and identify new candidate targets for therapeutic drugs (Mackay & Moore, 2014; Moore & Williams, 2009; Wei, Hemani, & Haley, 2014). Although a few remarkable epistatic interactions, such as the Bombay phenotype (Kelly et al., 1994), have been described in the medical genetics literature, researchers have had few successes to date finding novel human gene‐gene interactions from modern array or sequencing studies of meaningful effect size that can be replicated across multiple data sets (Hemani et al., 2014).

The general lack of success identifying and replicating gene‐gene interactions can be attributed to a number of factors including poor power, ambiguity regarding marker coding and interaction scale, confounding, measurement error, and population stratification (Aschard et al., 2012a; Cordell, 2009). Of these factors, poor power is likely the most significant. For a genome‐wide association study (GWAS) measuring one million markers, a total of $\left(\begin{array}{c} 1\times 10^{6}\\ 2 \end{array}\right)$ or $∼5\times 10^{11}$ two‐way interactions are possible. If a separate statistical test is performed for each of these candidate interactions, an enormous penalty on statistical power will be incurred due to multiple hypothesis correction (MHC). Even if the set of markers is significantly filtered prior to interaction testing (Greene, Penrod, Kiralis, & Moore, 2009), for example, by evaluating only single nucleotide polymorphisms (SNPs) with significant main effects, the impact of MHC on power can still be substantial.

For cases where gene‐gene interaction detection power is unacceptably low, even after marker filtering, a global interaction test may be a feasible analysis alternative. Although global tests provide just a general indication of the presence of interactions, they do offer much greater statistical power due to the lack of MHC and, importantly, can be utilized in a hierarchical analysis (Yekutieli, 2008) to test subsets of the markers or different endpoints prior to individual interaction tests. Although the use of global gene‐gene interaction tests has been previously explored (Dai et al., 2012a), the approaches taken in prior research have been limited to two‐stage methods in which separate tests are first performed for each potential interaction and then the interaction‐level *P*‐values are jointly tested, using a test like Fisher's method (Won, Morris, Lu, & Elston, 2009), against the null that all *P*‐values are insignificant, that is, have a standard uniform distribution. Although such a two‐stage approach can be successful, it has several important drawbacks: it is computationally expensive, the results are dependent on the form of the interaction‐level tests, and the test is subject to an inflated type I error rate if the interaction *P*‐values are correlated and that correlation is not accurately modeled and accounted for in the joint *P*‐value test.

To improve upon the existing two‐stage approach for global gene‐gene interaction testing, we have developed a novel, parametric global test, the global epistasis test (GET), that is based on an important recent result from random matrix theory concerning the distribution of the largest eigenvalue of certain functions of two sample covariance matrices (Johnstone, 2009). Our GET method supports the detection of gene‐gene interactions on a log‐odds scale relative to a binary phenotype or dichotomized quantitative trait by first estimating sample correlation matrices for the cases and the controls and then using a scaled and centered version of the largest eigenvalue of a function of these sample correlation matrices to test the null hypothesis that the population correlation matrix for cases is equal to the population correlation matrix for controls. As we demonstrate using simulation studies, the GET method provides superior type I error control and power as compared to the standard two‐stage global gene‐gene interaction test. To show the practical utility of the GET method, we analyzed the glaucoma GWAS data from the Glaucoma Gene Environment (GLAUGEN) study, a part of GENEVA consortium (Cornelis et al., 2010).

The remainder of this paper is organized as follows: Section 2 outlines our data assumptions, the statistical details of the GET method, and the framework used for evaluation, Section 3 contains the results from simulation studies and the analysis of the GLAUGEN GWAS data set, and a discussion and summary is included in Section 4.

## 2 METHODS

### 2.1 Data assumptions and interaction model

We assume that detection of gene‐gene interactions is performed on a collection of genetic markers, a binary phenotype or dichotomized quantitative trait, and clinical covariates captured for multiple independent subjects as part of a GWAS.

#### 2.1.1 Genetic markers

We assume there are *p* genetic markers, $G_{1},...,G_{p}$, measured on all *n* subjects. Specifically, it will be assumed that these are SNPs specified using additive coding. The measured values can be stored in an n×p matrix, G, where each element equals 0, 1, or 2 according to the number of variant alleles at marker *j* for subject *i*. Let the p×p matrices Σ and P represent the population covariance and correlation matrices for the *p* markers. If M is the mean‐centered and standardized version of G, then the unbiased estimate of the sample correlation matrix can be defined by the matrix $S=1/\left(n-1\right)M^{T}M$ whose elements are the Pearson correlation coefficients between markers *i* and *j*. Because the elements of S are the Pearson correlation coefficients between the genetic markers, that is, SNPs, they represent the genotypic linkage disequilibrium (LD) measure $\hat{\Delta }$ between each pair of SNPs (Wellek & Ziegler, 2009; Ziegler & König, 2010).

#### 2.1.2 Phenotype

We assume that a phenotype, Y, is measured on all *n* subjects. We specifically assume that Y is a binary phenotype, for example, indicator of disease case/control status, or a continuous phenotype discretized as a binary variable. The term phenotype will be used in this paper to describe both standard phenotypes as well as variables often described as endophenotypes (Gottesman & Gould, 2003). Measured values of the phenotype can be stored in an n×1 vector, y, where element $y_{i}$ represents the value of the phenotype for subject *i*.

#### 2.1.3 Covariates

We assume that *k* covariates, $C_{1},...,C_{k}$, are measured on all *n* subjects. Measured values can be stored in a single n×k matrix C.

#### 2.1.4 Phenotype‐based partitioning

To formulate our global gene‐gene interaction test, it is necessary to partition the genetic marker data based on the value of the binary phenotype or dichotomized continuous variable and, based on this data split, define partitioned versions of the population and sample covariance and correlation matrices. The genetic marker matrix G and the mean‐centered and standardized marker matrix M and the covariate matrix C can all be split into two submatrices according to the value of the binary phenotype Y.

Let *d* represent the number of subjects whose measured value of phenotype Y is 1, that is, $d=\sum_{i=1}^{n}y_{i}$. The partitioned genetic marker matrices can then be defined as the d×p matrix $G^{Y=1}$ or $G^{1}$ and the (n−d)×p matrix $G^{Y=0}$ or $G^{0}$, where $G^{1}$ contains the *d* rows of G corresponding to subjects whose Y phenotype value is 1 and $G^{0}$ contains the n−d rows of G not in $G^{1}$. As a concrete example, if the phenotype variable Y represents disease case/control status, then $G^{1}$ holds the genetic marker data for cases and $G^{0}$ holds the genetic marker data for controls. The partitioned covariate matrices, $C^{1}$ and $C^{0}$, can be defined analogously to $G^{1}$ and $G^{0}$. The partitioned mean‐centered and standardized genetic marker matrices, $M^{1}$ and $M^{0}$, are computed by mean centering and standardizing the partitioned marker matrices $G^{1}$ and $G^{0}$, respectively.

Let the population correlation relationships among the genetic markers, $G_{1},...,G_{p}$, within the subpopulations defined by the phenotype variable Y be defined by the matrices:

$$P^{Y=1}=P^{1},P^{Y=0}=P^{0}.$$

Let the sample correlation relationships among the genetic markers within the subpopulations be defined by

(1) $$\begin{array}{ccc} & & S^{Y=1}=S^{1}=\frac{1}{d-1}{\left(M^{1}\right)}^{T}M^{1}\\ & & S^{Y=0}=S^{0}=\frac{1}{n-d-1}{\left(M^{0}\right)}^{T}M^{0}. \end{array}$$

If interaction detection is being performed while controlling for a nonzero number of covariates, $C_{k}$, it is assumed that $P^{0},P^{1},S^{0}$, and $S^{1}$ represent the population and sample partial correlation matrices whose elements contain the correlation between each pair of genetic markers conditional on the values of the covariates, $C_{k}$.

#### 2.1.5 Gene‐gene interaction model

The proposed GET method is concerned with identifying statistical two‐way interactions between genetic markers relative to the phenotype Y. For binary phenotypes, we assume in the remainder of this paper that interactions represent a departure from additivity on log‐odds scale although the method also applies to interaction detection on an absolute risk scale. Such interactions can be statistically tested in the case of a two‐way interaction between markers $G_{a}$ and $G_{b}$ relative to binary phenotype *Y* using a logistic regression model of the following form:

(2) $$\begin{array}{ccc} logit(P\left(Y=1|G_{a},G_{b},C\right) & = & \beta_{0}+\sum_{i=1}^{k}\beta_{C_{i}}C_{i}+\beta_{G_{a}}G_{a}\\ & & +\beta_{G_{b}}G_{b}+\beta_{G_{a}G_{b}}G_{a}G_{b}. \end{array}$$

Given this model, the null hypothesis of no interaction between $G_{a}$ and $G_{b}$ can be specified as $H_{0}:\beta_{G_{a}G_{b}}=0$.

### 2.2 GET Method

Our proposed GET method for detecting interactions compares the sample correlation matrix for cases, $S^{1}$, with the sample correlation matrix for controls, $S^{0}$, with hypothesis testing based on an important theoretical result from random matrix theory concerning the test of the equivalence of two covariance matrices (Johnstone, 2009). An R (R Core Team, 2015) implementation of the GET method and a simple example equivalent to the analysis shown in Table 1 are available at http://www.dartmouth.edu/~hrfrost/GET.

**Table 1:** Partitioned sample correlation matrices for data simulated with five SNPs and an interaction between SNPs 1 and 2, that is, marker variables *G*_1_ and *G*_2_

Average $S^{1}$ (cases)						Average $S^{0}$ (controls)					
	*G*_1_	*G*_2_	*G*_3_	*G*_4_	*G*_5_		*G*_1_	*G*_2_	*G*_3_	*G*_4_	*G*_5_
*G*_1_	1	−	−	−	−	*G*_1_	1	−	−	−	−
*G*_2_	−**0.15**	1	−	−	−	*G*_2_	**0.16**	1	−	−	−
*G*_3_	0.05	0.04	1	−	−	*G*_3_	0.06	0.09	1	−	−
*G*_4_	0.06	0.05	0.07	1	−	*G*_4_	0.07	0.07	0.07	1	−
*G*_5_	0.06	0.05	0.05	0.09	1	*G*_5_	0.06	0.08	0.09	0.06	1

The mean case and control correlation coefficients for the first two SNPs are in bold.

#### 2.2.1 Hypothesis

Given the phenotype‐based partitioning of the data and correlation matrices defined in Section 2.1.4, a global test for gene‐gene interactions can be specified using the following null and alternative hypotheses:

(3) $$H_{0}:P^{1}=P^{0},H_{A}:P^{1}\neq P^{0}.$$

This null hypothesis asserts that the population correlation structure for the *p* genetic markers is identical for both subpopulations according to the phenotype Y. Because the elements of the sample correlation matrix are equal to the LD measure $\hat{\Delta }$ (Wellek & Ziegler, 2009; Ziegler & König, 2010), this null hypothesis equivalently asserts that the LD structure measured for the markers among cases is equal to the LD structure measured among controls. It is useful to contrast this test with the case‐only test of gene‐environment or gene‐gene interactions (Ziegler & König, 2010). For a single interaction, the case‐only test estimates the correlation between two genetic markers (or between a genetic marker and an environmental exposure) within just the cases and tests the null hypothesis that this case‐only correlation is 0. If the two markers are uncorrelated in the general population, the case‐only test correctly controls the type I error rate and is among the most powerful of all interaction detection methods (Clarke & Morris, 2010). By testing the equality of case and control correlation matrices, the GET method jointly tests the difference between the case and control correlation coefficients for all pairs of markers, that is, all potential gene‐gene interactions. Importantly, this test does not require the markers to be uncorrelated in the general population because the comparison is made between case and control sample correlation matrices.

As a simple illustration, Table 1 contains the average of 20 partitioned sample correlation matrices, $S^{1}$ and $S^{0}$, computed for simulated genotype data with five SNPs and just a single interaction between the first two SNPs (represented by variables *G*_1_ and *G*_2_). Each data set was simulated to contain five SNPs measured on 1,000 independent subjects with additive coding, a minor allele frequency (MAF) of 0.25, and inter‐SNP correlation of ∼0.1 (the SNPs were generated as correlated binomial variables from a Gaussian copula with ρ=0.1). The binary phenotype was generated according to the model: $\mathrm{logit}(P\left(Y=1|G\right)=-1+\text{log}\left(1.5\right)G_{1}+\text{log}\left(1.5\right)G_{2}+\text{log}\left(3\right)G_{1}G_{2}$. To reflect a case/control design, a large number of subjects were first simulated and then subsampled to create a data set with 500 cases and 500 controls. Given this model, the phenotype is associated with only the first two SNPs via both marginal and interaction effects. As expected, the difference between the mean case and control correlation coefficients is much larger (by nearly an order of magnitude: 0.31 vs. 0.019) for the first two SNPs than for any other marker pair. Importantly, the correlation for SNPs 1 and 2 deviates from the average estimated correlation between other SNP pairs among both cases and controls.

#### 2.2.2 Statistical significance

The GET method tests the null hypothesis $P^{1}=P^{0}$ against the alternative $P^{1}\neq P^{0}$ using results from random matrix theory first described by Johnstone in 2009 (Johnstone, 2008, 2009). As detailed by Johnstone, if there are two random vectors, $p_{1}$ and $p_{2}$, that have multivariate normal distributions with associated population covariance matrices $\Sigma_{1}$ and $\Sigma_{2}$, the test of $H_{0}:\Sigma_{1}=\Sigma_{2}$ versus $H_{A}:\Sigma_{1}\neq \Sigma_{2}$ can be based on the largest eigenvalue of a function of two sample covariance matrices for samples of *n*_1_ and *n*_2_ independent observations of the $p_{1}$ and $p_{2}$ random vectors. If the sample covariance matrices in this case are $S_{1}$ and $S_{2}$, then the test of this hypothesis, the so‐called greatest root test, is based on the largest eigenvalue, λ_1_, of ${\left(n_{1}S_{1}+n_{2}S_{2}\right)}^{-1}n_{2}S_{2}$.

As reported by Johnstone (2009), the distribution of λ_1_ of ${\left(n_{1}S_{1}+n_{2}S_{2}\right)}^{-1}n_{2}S_{2}$ under $H_{0}:\Sigma_{1}=\Sigma_{2}$ can be well approximated by the Tracy‐Widom law of order 1 distribution, *F*_1_, after the following transformation, centering, and scaling:

(4) $$\frac{\mathrm{logit}\left(\lambda_{1}\right)-\mu \left(p,n_{1},n_{2}\right)}{\sigma (p,n_{1},n_{2})}\overset{D}{\to }F_{1},$$

where the centering and scaling terms μ and σ are defined as:

μ(p,n1,n2)=2log tan ϕ+γ2σ(p,n1,n2)3=16(n1+n2−1)21sin2(ϕ+γ)sinϕsinγsin2γ2=min(p,n2)−0.5n1+n2−1, sin 2ϕ2= max (p,n2)−0.5n1+n2−1.

Johnstone's development of this Tracy‐Widom approximation to the null distribution of the greatest root statistic was motivated largely by the very poor type I error control achieved by the standard distributional approximation, based on an *F* distribution (Johnstone, 2009).

For the GET method, the test statistic is based on the principal eigenvalue of a similar matrix computed using the partitioned sample correlation matrices, **S** ^1^ and **S** ^0^: ${\left(dS^{1}+\left(n-d\right)\;S^{0}\right)}^{-1}\left(n-d\right)\;S^{0}$. To generate a test statistic, *T*, that can be used for testing the hypothesis specified in (3), the principal eigenvalue of this function of **S** ^1^ and **S** ^0^ is computed and then the transformation, centering, and scaling specified in (4) are applied with $n_{1}=d$ and $n_{2}=n-d$:

(5) $$\begin{array}{c} T\;=\;\frac{\mathrm{logit}(\lambda_{1}\left({\left(dS^{1}\;+\;\left(n\;-\;d\right)S^{0}\right)}^{-1}\left(n\;-\;d\right)S^{0}\right)\;-\;\mu \left(p,d,n\;-\;d\right)}{\sigma (p,d,n\;-\;d)}. \end{array}$$

The *P*‐value associated with hypothesis (3) is therefore based on the probability that a Tracy‐Widom law of order 1 distribution is greater than *T*:

(6) $$Pr\left(T=t|H_{0}\right)=1-F_{1}\left(t\right).$$

**Figure 1:**
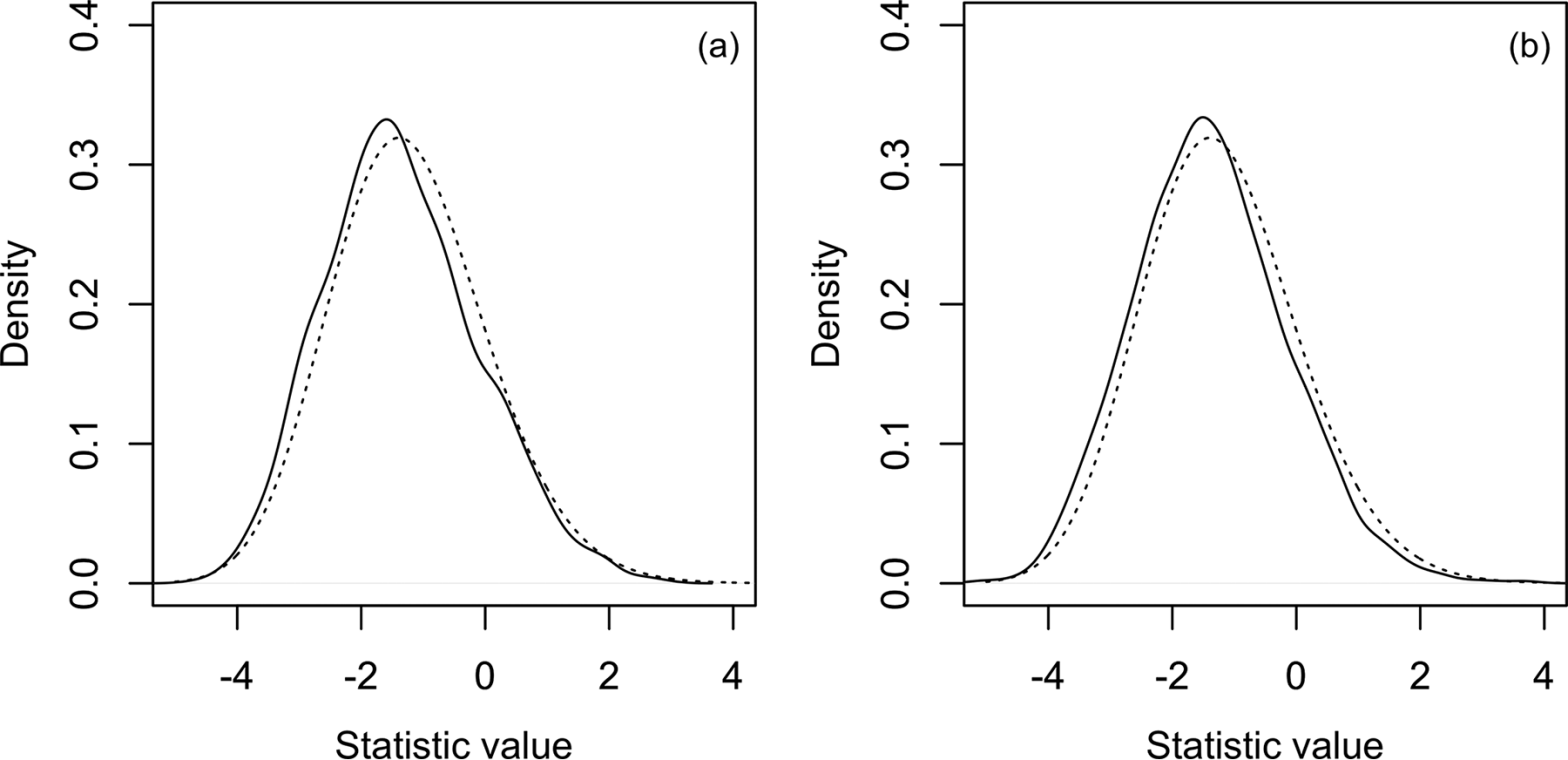
Comparison of the empirical density of the test statistic T defined in (5) (solid line) and the Tracy‐Widom law of order 1 distribution (dotted line). In plot (a), the T density was computed from 2,500 simulated data sets each containing 100 SNPs without marginal or interaction effects (see text in Section 2.2.2 for simulation details). In plot (b), the T density was computed from 2,500 simulated data sets each containing 100 SNPs with no interaction effects and a marginal association for the first five SNPs.

A potential limitation of using (6) to compute the *P*‐value is the fact that Tracy‐Widom distribution in (4) is asymptotic (n→∞ and p→∞ with n/p constant) and technically applies to just multivariate normal data while $S^{1}$ and $S^{0}$ are sample correlation matrices for binomial data measured on a finite number of observations. The centering and scaling terms μ and σ defined by Johnstone are also just approximations. However, it has been found that the Tracy‐Widom distribution associated with the largest eigenvalue of Wishart matrices actually hold quite well for non‐normal data as well as for small *n* and small *p* (Patterson, Price, & Reich, 2006; Soshnikov, 2002). To validate the accuracy of the Tracy‐Widom law of order 1 distribution for characterizing the statistic T defined in (5), we simulated two collections of 2,500 null data sets with each data set containing 500 subjects and 100 SNPs with MAF of 0.25, no intermarker correlation and no interactions. In the first collection of 2,500 data sets, none of the SNPs had marginal or interaction effects while in the second collection five of the 100 SNPs had a marginal association. As shown in Figure 1, the null distribution of the T statistic is well approximated by the Tracy‐Widom law of order 1 (as generated by the R function dtw in package RMTstat (Johnstone, Ma, Perry, & Shahram, 2014)) for both the case of no marginal or interaction effects and for the case of just marginal effects. Consistent with the results reported by Johnstone (2009) for the distributional approximation (4), the deviation between the empirical density of T and the Tracy‐Widom law of order 1 is in the conservative direction.

#### 2.2.3 Handling high‐dimensional data

If the number of markers *p* is relatively small (i.e., thousands to tens of thousands) computation of partitioned sample covariance matrices $S^{0}$ and $S^{1}$ as defined in (1) and the matrix ${\left(dS^{1}+\left(n-d\right)\;S^{0}\right)}^{-1}\left(n-d\right)\;S^{0}$ will be straightforward. For typical GWAS data sets, however, there will often be hundreds of thousands to millions of markers and, for such data sets, two serious challenges are encountered. First, the computation of the $S^{0}$ and $S^{1}$ matrices becomes computationally expensive, and second, the ratio of the number of samples to the number of markers (n/p ) becomes small, degrading the accuracy of the correlation matrix estimates and of distributional approximation (4). To support such genome‐scale data sets, the set of markers must therefore be filtered according to either biological or statistical criteria. Biological filtering can be done at the level of individual genes, that is, test for interactions among all markers proximate to coding regions for a specific gene, or at the level of entire pathways, that is, test for interactions among all markers associated with the genes involved in a specific pathway or other biologically based gene set (e.g., the gene sets defined in a repository such as MSigDB (Liberzon et al., 2011)). For statistical filtering, the set of markers is filtered according to some statistic computed from the data with the goal of just keeping markers that have a high likelihood of association with the outcome of interest. This type of filtering, often called screening‐testing (Bourgon, Gentleman, & Huber, 2010), is commonly used during interaction detection to reduce the burden of MHC (Murcray, Lewinger, & Gauderman, 2009; Murcray, Lewinger, Conti, Thomas, & Gauderman, 2011). For screening‐testing, the filter statistic must be independent from the interaction test statistic under the null hypothesis to ensure type I error control using the unfiltered markers. For the simulation studies detailed in Sections 2.3.2 and 2.3.3, the number of markers was set to a size that could be reasonably achieved via either biological or statistical filtering. For the real data use case detailed in Section 2.3.4, statistical filtering was employed with the marginal association between each marker and the phenotype as a filter statistic, that is, the *P*‐value associated with ${\hat{\beta }}_{G_{a}}$ in (2). Our selection is motivated by the fact that the marginal association filter statistic is asymptotically independent from the gene‐gene interaction test statistic under the null hypothesis of no interaction (Dai, Kooperberg, Leblanc, & Prentice, 2012b).

### 2.3 Evaluation design

To evaluate our GET method, we compared the results from GET for simple simulation (Section 2.3.2), disease‐based simulation (Section 2.3.3), and real data (Section 2.3.4) use cases against the benchmark approach outlined in Section 2.3.1.

#### 2.3.1 Benchmark global interaction test

For comparative evaluation of the GET method, we used the best performing global interaction detection method in Dai et al. (2012a). In Dai et al., a two‐stage global test was performed in which a *P*‐value was first computed for each potential interaction and then the set of interaction *P*‐values were tested for departure from the *U*(0, 1) distribution expected under the null hypothesis of no gene‐gene interactions. Dai et al. evaluated multiple methods for performing this combined *P*‐value test and found that Fisher's method (Kost and McDermott, 2002; Won et al., 2009) had the best overall performance.

In Dai et al., the multifactor dimensionality reduction (MDR) method (Moore, 2010) was used to compute interaction‐level *P*‐values. For the simulation‐based evaluation of the GET method, we used the case‐only trend test for association between the two genetic markers to compute interaction‐level *P*‐values. The test is based on the following linear regression model:

$$E\left(G_{a}|Y=1,G_{b},C\right)=\beta_{0}+\sum_{i=1}^{k}\beta_{C_{i}}C_{i}+\beta_{G_{b}}G_{b}$$

with the null hypothesis that $\beta_{G_{b}}=0$. Statistical significance can be computed via a Wald test on ${\hat{\beta }}_{G_{b}}$ or using a likelihood ratio test. We selected this test to achieve a competitive power comparison and to highlight issues with type I error control. As detailed in Clark et al. (Clarke & Morris, 2010), the case‐only trend test has the best overall power among interaction detection tests when specific genetic architecture, for example, dominance, cannot be assumed. As a case‐only test, it can also generate highly inflated type I error rates when genetic markers are correlated in the general population.

For the GLAUGEN GWAS analysis detailed in Section 2.3.4, the *P*‐values for individual SNP‐SNP interactions were computed using the case‐only version of PLINK's “fast‐epistasis” test (Chang et al., 2015).

#### 2.3.2 Simple simulation design

The type I error control and power of the GET method were assessed relative to the benchmark approach using a simple simulation model based on the framework used in Dai et al. (2012b). This simple model has the benefits of easy interpretation and straightforward implementation by other researchers.

For type I error control assessment, we performed simulations for 20 different groups of parameter settings. Specifically, 1,000 data sets were simulated with 250, 500, 1,000, 2,000, or 4,000 independent subjects, 50 SNPs generated with additive coding under an assumption of Hardy‐Weinberg equilibrium with a MAF randomly selected from *U*(0.25, 0.75), and a correlation between SNPs of either 0 or ∼0.1 (the SNPs were simulated as correlated binomial variables from a Gaussian copula with ρ=0 or ρ=0.1). A single binary phenotype was generated according to model (2) with no interaction effects (i.e., $\beta_{G_{i},G_{j}}=0$) and the intercept and marginal association coefficients set to either: $\beta_{0}=-5$ and $\beta_{G_{i}}=0$ or $\beta_{0}=-2$ and $\beta_{G_{i}}=\text{log}\left(1.15\right),i=1,...,10$. The 20 simulated parameter settings included all possible combinations of the number of subjects, inter‐SNP correlation, and phenotype model coefficient settings. Similar to the simulation approach used to generate the results in Table 1 and the simulation support in tools like PLINK 1.9 (Chang et al., 2015) and GCTA (Yang, Lee, Goddard, & Visscher, 2011), a large number of subjects were generated that were then subsampled to ensure an even split between cases and controls.

For a comparative assessment of statistical power, we performed simulations for 15 different groups of parameter settings. Similar to the type I error control simulations, 1,000 data sets were simulated for each group of parameter settings with the number of subjects, number of SNPs, and SNP MAF taking the same range of values used for the type I error control simulations. Because the benchmark method is unable to maintain type I error control in the presence of inter‐SNP correlation, all SNPs were independent for the power simulation. The binary phenotype was also generated according to model (2) but, in this case, interaction effects were included for five of the potential SNP‐SNP pairs. Specifically, the following three different model coefficient settings were used:

1. *Main effects, interactions among main effect SNPs*: $\beta_{0}=-3$, $\beta_{G_{i}}=\text{log}\left(1.15\right),i=1,...,10$ and $\beta_{G_{i},G_{j}}=\text{log}\left(2\right),i,j\in \left(1,5\right)$ (i.e., marginal effects for the first five SNPs and interactions for five random SNP pairs drawn from the first five SNPs).
2. *Main effects, interactions among nonmain effect SNPs*: $\beta_{0}=-3$ and $\beta_{G_{i}}=\text{log}\left(1.15\right),i=1,...,10$ and $\beta_{G_{i},G_{j}}=\text{log}\left(2\right),i,j\notin \left(1,5\right)$ (i.e., marginal effects for the first five SNPs and interactions for five random SNP pairs drawn from all SNPs not including the first five).
3. *No main effects, interactions among nonmain effect SNPs*: $\beta_{0}=-1$ and $\beta_{G_{i}}=0$ and $\beta_{G_{i},G_{j}}=\text{log}\left(2\right)$ (i.e., no marginal effects and interactions for five random pairs of SNPs).

The 15 simulated parameter settings included all possible combinations of the number of subjects and phenotype model coefficient settings. Power was computed as the proportion of the 1,000 simulated data sets with a global gene‐gene interaction test *P*‐value below 0.05.

#### 2.3.3 Disease‐based simulation design

To assess the type I error control and power of GET in a more realistic scenario, we used the approach of Aschard et al. (2012a) for simulating gene‐gene interactions under the genetic architectures of breast cancer, type 2 diabetes, and rheumatoid arthritis. Specifically, we modified Aschard's original simulation code to generate disease‐based data sets with or without G× G interactions. For both type I error control and power evaluation, 1,000 data sets were generated for each disease with each data set containing either 625, 1,250, or 2,500 subjects with data for the set of known risk SNPs and known environmental risk factors, as detailed for each disease in the Supporting Information for Aschard et al. (2012a), and a set of 50 additional SNPs with a MAF drawn from *U*(0.05, 0.95) and no association with the outcome. No G× E interactions were included and no correlation was simulated between markers, between exposures or between markers and exposures. The binary outcome variable, reflecting disease case‐control status, was simulated according to the model represented by Equation (2) in Aschard et al. (2012a). Similar to the approach used for the simple simulation model, we mimicked case‐control study data by simulating a large number of subjects and then sampling cases and controls to achieve an equal case/control balance. For type I error control simulation, no G× G interactions were simulated. For power simulation, five G× G interactions were simulated among randomly selected pairs of the known risk SNPs for each disease with the interaction effect in the Aschard model set to ±log(2).

#### 2.3.4 GLAUGEN GWAS analysis

To assess the practical utility of the GET method, we analyzed GWAS data from the GLAUGEN study, a part of GENEVA consortium (Cornelis et al., 2010) now combined with the NEIGHBOR (NEI Glaucoma Human genetics collaBORation) study into the NEIGHBOR consortium (Wiggs et al., 2013). The GLAUGEN GWAS aims to characterize genetic markers and gene‐environment interactions associated with primary open‐angle glaucoma (POAG), the most common type of glaucoma worldwide (the prevalence among Caucasian individuals over 70 years of age is 6% (Rudnicka, Mt‐Isa, Owen, Cook, & Ashby, 2006)) and a disease with significant heritability (Sung et al., 2006). POAG is thought to be caused by a slow exit of aqueous humor through the trabecular meshwork leading to a progressive buildup of fluid, increased intraocular pressure (IOP), and damage to the optic nerve (Llobet, Gasull, & Gual, 2003). The combined NEIGHBOR consortium contains nearly 3,500 cases and 3,500 controls, limited to individuals 35 years of age or older with European‐derived or Hispanic Caucasian ethnicity.

For our analysis, we used the subset of the NEIGHBOR consortium GWAS data that was drawn from the GLAUGEN study: 1,000 cases and 1,183 controls. In addition to POAG diagnosis, we considered the following ocular endophenotypes: VFPA (indicator of paracentral vision loss), VFPE (indicator of peripheral vision loss), IOP (maximum untreated interocular pressure), VFPSD (pattern standard deviation), VCDR (recent vertical cup/disk ratio). For all of the endophenotypes, we adopted a simple two eye design in which the average of the right and left eye measurements was used and, if one eye measurement was missing, then the nonmissing value was used (Glynn & Rosner, 2012; Murdoch, Morris, & Cousens, 1998). If a binary endophenotype had a positive indicator in just one eye, this was interpreted as a positive average. For the continuous endophenotypes IOP, VFPSD, and VCDR, median‐based dichotomization of the right and left eye averages was used to create a binary phenotype (values equal to the median were treated as cases).

Quality control of the SNP data was carried out using PLINK 1.9 (Chang et al., 2015) and included removing subjects missing more than 5% of the SNPs, removing all nonautosomal SNPs, removing all SNPs with a Hardy‐Weinberg test of equilibrium *P*‐value $<1\times 10^{-5}$, removing all SNPs with MAF of less than 0.01 and removing all SNPs with any missing measurements. Although very conservative, this last QC step eliminated the potential bias of an imputation method and was considered appropriate because the aim of the analysis was not to maximize the number of significant findings but instead support the comparative evaluation of GET with the benchmark method on real GWAS data. After all of these preprocessing and quality control steps, 2,112 subjects (976 cases and 1,136 controls) and 200,432 SNPs, specified using additive coding, remained in the data set. Because of missing endophenotype values, the actual number of subjects available for analysis of each endophenotypes varied as follows (with case/control in this context referring to the dichotomized value of the endophenotype): VFPA: 127 cases, 510 controls; VFPE: 357 cases, 175 controls; IOP: 624 cases, 549 controls; VFPSD: 432 cases, 433 controls; VCDR: 678 cases, 606 controls.

Given the large number of SNPs remaining after QC (200,432), we performed filtering using the marginal association filter statistic, as outlined in Section 2.2.3, prior to gene‐gene interaction detection For continuous phenotypes, the marginal association filter statistic was computed via linear regression; for the binary phenotypes, it was computed using logistic regression. The filter threshold for each phenotype was adjusted to retain 100 SNPs for the final analysis (for an n/p∼20 and 1002=4,950 potential SNP‐SNP interactions).

False discovery rate (FDR) values were computed for global interaction test *P*‐values and for the specific SNP‐SNP interaction test *P*‐values using the Benjamini and Hochberg method (Benjamini & Hochberg, 1995). For global interaction *P*‐values, the family of hypotheses for FDR computation comprised the tests associated with the six phenotypes listed above. For each of these phenotypes, the family of hypotheses for FDR computation comprised the gene‐gene interaction tests for all 4,950 potential SNP‐SNP interactions. For this analysis, we did not adjust the interaction‐level FDR values to account for global tests. If strict control of the FDR was desired for the interaction‐level tests, the hierarchical FDR approach of Yekutieli (2008) could be employed.

## 3 RESULTS

### 3.1 Simple simulation results

#### 3.1.1 Type I error control

As shown in Table 2, type I error control for the GET method was conservative for all tested simulation settings. This result is consistent with the findings by Johnstone (2009) that the Tracy‐Widom approximation was conservative for all simulations settings shown in his Table 1 except the simulation with the smallest n/p ratio (n/p=∼3) for which type I error control was anticonservative. The type I error rate for GET was insensitive to the presence of a general correlation between SNPs or the presence of a marginal association for some of the SNPs. For the benchmark method, type I error control was closely associated with the overall correlation between the simulated SNPs. When the SNPs were simulated without any correlation, type I error control was excellent, however, even a small level of correlation between SNPs resulted in a serious inflation of the type I error rate. Because the individual interaction *P*‐values were computed using a case‐only test and the joint *P*‐value test assumed *P*‐value independence, this result was expected.

**Table 2:** Results for the type I error control simulation as detailed in Section 3.1

Simulation settings			Type I error rate	
Marginal assoc.	(No. of subjects)/(No. of SNPs)	SNP‐SNP ∼ρ	GET	Benchmark method
✓	250/50=5	0.0	0.044	0.056
✓	500/50=10	0.0	0.024	0.044
✓	1,000/50=20	0.0	0.015	0.046
✓	2,000/50=40	0.0	0.017	0.049
✓	4,000/50=80	0.0	0.017	0.052
✓	250/50=5	0.1	0.033	1.000
✓	500/50=10	0.1	0.029	1.000
✓	1,000/50=20	0.1	0.025	1.000
✓	2,000/50=40	0.1	0.026	1.000
✓	4,000/50=80	0.1	0.018	1.000
	250/50=5	0.0	0.035	0.047
	500/50=10	0.0	0.018	0.042
	1,000/50=20	0.0	0.027	0.050
	2,000/50=40	0.0	0.014	0.044
	4,000/50=80	0.0	0.019	0.060
	250/50=5	0.1	0.039	1.000
	500/50=10	0.1	0.025	1.000
	1,000/50=20	0.1	0.023	1.000
	2,000/50=40	0.1	0.024	1.000
	4,000/50=80	0.1	0.017	1.000

#### 3.1.2 Power

As seen in Table 3, the GET method had superior power relative to the benchmark method for almost all of the simulation settings with n/p≥20. As expected, power improved for both methods as the n/p ratio increased with the relative difference in power between GET and the benchmark method becoming substantial for n/p≥40. Neither method was able to detect the simulated interactions when n/p≤10. Power for GET was also found to increase as both *n* and *p* increased with the ratio n/p held constant (results not shown), which is consistent with the improved quality of the Tracy‐Widom approximation as both *n* and *p* increase with the ratio n/p fixed. For GET, performance was similar across all three models while the benchmark method displayed significant model sensitivity with significant drops in empirical power between models 1 and 2 and between models 2 and 3.

**Table 3:** Results for the power simulation as detailed in Section 2.3.2.

Simulation settings		Power	
Model no.	(No. of subjects)/(No. of SNPs)	GET	Benchmark method
1	250/50=5	0.039	0.056
1	500/50=10	0.056	0.066
1	1,000/50=20	0.086	0.120
1	2,000/50=40	0.304	0.165
1	4,000/50=80	0.817	0.335
2	250/50=5	0.055	0.060
2	500/50=10	0.052	0.069
2	1,000/50=20	0.094	0.090
2	2,000/50=40	0.344	0.123
2	4,000/50=80	0.921	0.270
3	250/50=5	0.048	0.064
3	500/50=10	0.044	0.057
3	1,000/50=20	0.108	0.055
3	2,000/50=40	0.469	0.089
3	4,000/50=80	0.945	0.121

In the table, model no. refers to one of the numbered simulation models detailed in Section 2.3.2.

### 3.2 Disease‐based simulation results

As seen in Table 4, the GET method had acceptable type I error control for all three simulated disease architectures detailed in Section 2.3.3 at each of the tested sample sizes. The benchmark method, on the other hand, exhibited conservative type I error control in all evaluated cases. The empirical power realized by GET was significantly higher than the power for the benchmark method for all disease architectures and sample sizes. The benchmark method had only marginal power at the very largest sample size. As expected, the power for both methods increased as the number of subjects was increased.

**Table 4:** Estimated type I error rates at empirical power at α=0.05 the disease‐based simulation studies detailed in Section 2.3.3.

Disease model	No. of subjects	Method	Type I error rate	Power
Breast cancer	625	Benchmark	0.038	0.042
	625	GET	0.058	0.466
	1,250	Benchmark	0.015	0.060
	1,250	GET	0.052	0.893
	2,500	Benchmark	0.031	0.225
	2,500	GET	0.062	1.000
Type 2 diabetes	625	Benchmark	0.032	0.036
	625	GET	0.040	0.202
	1,250	Benchmark	0.027	0.048
	1,250	GET	0.042	0.400
	2,500	Benchmark	0.018	0.143
	2,500	GET	0.063	0.806
Rheumatoid arthritis	625	Benchmark	0.028	0.034
	625	GET	0.048	0.279
	1,250	Benchmark	0.024	0.049
	1,250	GET	0.051	0.619
	2,500	Benchmark	0.025	0.124
	2,500	GET	0.058	0.965

### 3.3 GLAUGEN results

Table 5 contains the global gene‐gene interaction test results generated by GET and the benchmark method for GLAUGEN GWAS data using six different phenotypes, as detailed in Section 2.3.4. Using the GET method, highly significant FDR values were generated for all of the phenotypes with the exception of paracentral vision loss. Using the benchmark method, only three of the phenotypes had significant global gene‐gene interaction test results at a q≤0.1: peripheral vision loss, pattern standard deviation, and recent vertical cup/disk ratio. Importantly, the benchmark method failed to detect evidence of gene‐gene interactions relative to either the POAG diagnosis or maximum untreated IOP, one of the key diagnostic indicators of glaucoma.

**Table 5:** Global gene‐gene interaction detection results for the GLAUGEN GWAS data using GET and the benchmark method using the procedure detailed in Section 2.3.4.

Phenotype	No. of cases	No. of controls	GET FDR	Benchmark FDR
Primary open‐angle glaucoma (POAG)	976	1,136	0.0094	0.175
Paracentral vision loss (VFPA)	127	510	0.414	0.853
Peripheral vision loss (VFPE)	357	175	∼0	0.0073
Maximum untreated intraocular pressure (IOP)	624	549	$1.319\times 10^{-21}$	0.464
Pattern standard deviation (VFPSD)	432	433	∼0	0.0018
Recent vertical cup/disk ratio (VCDR)	678	606	0.00094	0.0128

Table 6 displays the significant SNP‐SNP interactions at q≤0.1 for each of phenotypes for which GET generated a significant global test result at q≤0.1. Only four of the five phenotypes with significant global interaction tests according to GET had significant SNP‐SNP interactions among the 100 SNPs kept after marginal association filtering. In this case, each of the four phenotypes had just a single significant SNP‐SNP interaction. Although only a small number of significant interactions were found, this result was consistent with the limited interaction detection power for this analysis. Specifically, interaction detection power was limited by the stringent filtering of SNPs by marginal association, the relatively small sample size, the impact of MHC for the family of 4,950 potential interactions after filtering, and other factors such as ambiguity regarding marker coding and interaction scale, measurement error, and confounding (Aschard et al., 2012b). As far as we are aware, none of these interactions have been reported before in the literature. Furthermore, none of the SNPs in these significant interactions have reported GWAS associations in the NHGRI‐EBI GWAS Catalog (Welter et al., 2014) and none of the related genes contain markers with significant associations to glaucoma‐related phenotypes. Based on previously published associations for the genes linked to the SNPs, the interactions for IOP, VFPSD, and VCDR all represent biologically plausible findings for a glaucoma GWAS study and merit future investigation.

**Table 6:** SNP‐SNP interactions at a level‐specific FDR q≤0.1 according to the interaction testing method detailed in Section 2.3.1.

Phenotype	SNP 1 (gene)	SNP 2 (gene)	FDR
VFPE	rs13396549	rs9863361	0.0012
	(PARD3B)	(∼3 kb from ncRNA LOC105734230)	
IOP	rs10246477	rs12324434	0.082
	(SEMA3E)	(DYX1C1)	
VFPSD	rs2419666	rs7914325	0.021
	(∼6 kb from CNV nsv995491)	(ABLIM1)	
VCDR	rs481154	rs11154524	0.029
	(DNM3)	(SAMD3)	

No significant SNP‐SNP interactions were found at q≤0.1 for the primary open‐angle glaucoma (POAG) or paracentral vision loss (VFPA) phenotypes. For each SNP in the interactions, the rs number and associated gene, if one exists according to dbSNP, are listed. If no gene association exists for the SNP in dbSNP, the closest gene is indicated.

For the rs10246477‐rs12324434 interaction, there is experimental support for the association between the two SNP‐associated genes, SEMA3E and DYX1C1, and IOP. SEMA3E (semaphorin 3E), one of a large family of semaphorin protein coding genes, uses plexin and neurtrophilin coreceptor signaling to regulate vascular patterning (Aghajanian et al., 2014). Of direct relevance to this analysis, SEMA3E has been identified as an important regulator of vascular network development (Kim, Oh, Gaiano, Yoshida, & Gu, 2011) and mutations in SEMA3E may be associated with megalocornea (Davidson et al., 2014). It is therefore plausible that a mutation in SEMA3E could result in alterations of the retinal vascular network that increase the risk for high IOP due to poor drainage of the aqueous humor. The DYX1C1 gene has been linked to M(y)‐cell‐based magnocellular dysfunction (Pammer & Wheatley, 2001). Importantly, M(y) cells are lost early in glaucoma and M‐cell loss has been found in glaucoma experimentally induced in monkeys by damaging the trabecular meshwork to increase IOP (Crawford, Harwerth, Smith, Shen, & Carter‐Dawson, 2000). For the rs2419666‐rs7914325 interaction, both the gene associated with rs7914325 (ABLIM1) and the copy number variant (CNV) adjacent to rs2419666 (nsv995491) are associated with biological functions linked to POAG. Specifically, the ABLIM1 gene is related to changes in the actin cytoskeleton and is associated with POAG and steroid‐induced glaucoma via cytoskeletal changes in trabecular meshwork cells (Clark et al., 2013). The CNV adjacent to SNP rs2419666 (nsv995491) is related to a number of skeletal and craniofacial phenotypes (dolichecephaly, micrognathia, and pectus excavatum) that can co‐occur with structural defects in the eye, for example, Marfan's syndrome is associated with a number of ocular phenotypes (Latasiewicz, Fontecilla, Millá, & Sánchez, 2016) including glaucoma (Izquierdo, Traboulsi, Enger, & Maumenee, 1992). For the rs481154‐rs11154524 interaction, both SNP‐associated genes, SAMD3 and DNM3, again have known glaucoma associations. Specifically, an experiment using SAMD3 knockout mice found that SAMD3 is a required signaling protein for TGFβ2 controlled expression of ECM proteins in tabecular meshwork cells leading to increased IOP (McDowell, Tebow, Wordinger, & Clark, 2013). DNM3, a member of the larger family of dynamin motor proteins, has been found to play a functional role in the development of megakaryocyte cells (Wang, Gilligan, Sun, Wu, & Reems, 2011) and it is hypothesized that hematopoietic cells such as megakaryocytes are involved in the vascular events leading to POAG (Knepper & Samples, 2016). Although the potential biological mechanisms underlying the identified interactions are unclear, the fact that all of the SNP‐associated genes have been linked to biological processes important to the etiology of POAG provides strong motivation for further experimental exploration.

In terms of a comparative analysis, use of the GET method in a two‐stage analysis enabled the identification of three plausible SNP‐SNP interactions for the IOP, VFPSD, and VCDR phenotypes. The benchmark method, on the other hand, was only able to identify the SNP‐SNP interactions for the VFPSD and VCDR phenotypes.

## 4 DISCUSSION

Biologically important statistical interactions between markers of genetic variation, or gene‐gene interactions, are believed to exist for many complex phenotypes in numerous organisms (Cordell, 2009; Mackay, 2014; Moore, 2003; Taylor & Ehrenreich, 2015), however, despite the significant research effort, very few interactions of meaningful effect size have been discovered and replicated (Hemani et al., 2014). Although numerous issues impede gene‐gene interaction discovery, the greatest challenge is likely poor statistical power due to the burden of MHC (Aschard et al., 2012a; Cordell, 2009). Although prefiltering of the markers can improve interaction detection power by limiting the number of tested hypotheses (Greene et al., 2009), for many data sets, power remains insufficient to detect individual interactions.

For cases where individual interaction testing is not feasible due to low power, the use of a single global test for interactions is an attractive alternative. By eliminating MHC, global tests dramatically improve power relative to single interaction tests and can be used to enable a multistage or hierarchical analysis in which multiple phenotypes or marker subsets are first tested via global tests with individual interaction testing only performed for cases with a significant global test. Although work on global gene‐gene interaction testing has been explored by previous researchers, for example, Dai et al. (2012a), approaches have so far been limited to two‐stage methods in which a separate statistical test is first performed for all potential interactions and then the set of interaction *P*‐values are jointly tested using a test such as Fisher's method against the null that they are all insignificant.

To address the limitations of two‐stage global gene‐gene interaction methods, we have developed a novel, parametric global interaction test, the GET, based on an important result from random matrix theory. As detailed in Section 2.2, our GET method partitions the genetic marker data according to a binary phenotype or dichotomized quantitative trait and tests the null hypothesis that the population correlation matrix for cases is equal to the population correlation matrix for controls. This test is accomplished by transforming the largest eigenvalue of a function of the sample correlation matrix for cases and the sample correlation matrix for controls to a statistic that has a null distribution well approximated by the Tracy‐Widom law of order 1 (Johnstone, 2009). Because the elements of the partitioned sample correlation matrices represent the genotypic LD measure $\hat{\Delta }$ between each pair of markers (Wellek & Ziegler, 2009; Ziegler & König, 2010), the null hypothesis can also be viewed as asserting that the LD measure $\hat{\Delta }$ for each pair of markers is the same among cases and controls.

As we demonstrated via simple and disease‐based simulation studies (detailed in Sections 2.3.2 and 2.3.3 with results in Sections 3.1 and 3.2), the GET method has superior type I error control and power relative to a benchmark two‐stage method based on the best approach tested by Dai et al. (2012a). Regarding type I error control, the GET method has the important advantage, relative to the benchmark method, of general insensitivity to correlation among markers. As seen in Table 2, two‐stage methods can generate highly inflated type I error rates when markers are correlated. In terms of statistical power, the GET method was found to be more powerful than the benchmark method across a wide range of simulation settings (see Tables 3 and 4) with power increasing as the ratio of subjects (*n*) to markers (*p*) increased.

To explore the practical utility of the GET method, we analyzed the GLAUGEN glaucoma GWAS data (Cornelis et al., 2010), specifically looking for SNP‐SNP interactions relative to a set of six different measured phenotypes. As detailed in Section 3.3 and Tables 5 and 6, the GET method returned significant global test results for five of the six phenotypes with four of the five significant phenotypes having a single significant SNP‐SNP interaction. Importantly, three of the four significant SNP‐SNP interactions are biologically plausible based on published associations for the related genes and, to the best of our knowledge, have not been previously reported in the literature. For glaucoma researchers, these significant and novel SNP‐SNP interactions, and the associated genes, may offer new insights into the etiology of glaucoma and possible molecular targets for therapeutic intervention. These findings are even more impressive given the fact that the GLAUGEN analysis was structured for a comparative analysis and not to maximize the chance of interesting findings. In contrast to GET, the benchmark method only returned significant global test results for three of the six phenotypes and thus only detected two of the three biologically plausible interactions found via GET. In the likely scenario that a researcher started the GLAUGEN analysis by performing a global interaction test relative to just the POAG phenotype, use of the benchmark method would have failed to find any significant SNP‐SNP interactions, whereas a researcher employing the GET method may have been motivated to explore other phenotypes given the significant global test result, but lack of individually significant interactions, for POAG.

Some important limitations of the GET method should be noted. A key limitation of GET is poor statistical power at low values of n/p. Although this lack of power at low n/p is shared by other available methods, it does necessitate some form of biological or statistical prefiltering of markers for most realistic data sets. Another important limitation of the GET method is that it requires a binary phenotype. In the event that a continuous phenotype is measured, discretization to support GET will generally result in decreased statistical power. Although GET can support covariate adjustment, this requires the use of partial correlation matrices that complicates the computational process. The fact that GET is a global test must also be reiterated. Because the null hypothesis for a global test is that no gene‐gene interactions exist, such a test can only provide an indication of whether any interactions exist within a data set; global tests cannot identify specific interactions. Directions for future research on the GET method include a theoretical investigation of the statistical properties of the test at small n/p values, the use of the T statistic defined in (5) to estimate the proportion of phenotypic variance due to epistasis and the use of the method to test subsets of markers based on biological pathways, for example, test sets of markers associated with the genes annotated to different biological pathways.

In summary, the GET method represents an important advance for gene‐gene interaction detection. Relative to the standard two‐stage approach for global G× G detection, the GET method provides superior power across a diverse range of models and, importantly, maintains type I error control in the presence of general intermarker correlation. As demonstrated by the GLAUGEN glaucoma GWAS analysis, the GET method can be leveraged to make novel and biologically important G× G findings that would be undetected using current techniques.
